# Cross-validated tree-based models for multi-target learning

**DOI:** 10.3389/frai.2024.1302860

**Published:** 2024-02-16

**Authors:** Yehuda Nissenbaum, Amichai Painsky

**Affiliations:** Department of Industrial Engineering, Tel Aviv University, Tel Aviv, Israel

**Keywords:** classification and regression trees, multi-target learning, tree-based models, gradient boosting, random forest

## Abstract

Multi-target learning (MTL) is a popular machine learning technique which considers simultaneous prediction of multiple targets. MTL schemes utilize a variety of methods, from traditional linear models to more contemporary deep neural networks. In this work we introduce a novel, highly interpretable, tree-based MTL scheme which exploits the correlation between the targets to obtain improved prediction accuracy. Our suggested scheme applies cross-validated splitting criterion to identify correlated targets at every node of the tree. This allows us to benefit from the correlation among the targets while avoiding overfitting. We demonstrate the performance of our proposed scheme in a variety of synthetic and real-world experiments, showing a significant improvement over alternative methods. An implementation of the proposed method is publicly available at the first author's webpage.

## 1 Introduction

Multi-target learning (MTL) is a supervised learning paradigm that aims to construct a predictive model to multiple response variables from a common set of features. This paradigm is also known as multi-variate (Brown and Zidek, 1980; Breiman and Friedman, 1997) or multi-output learning (Liu et al., 2009; Yao et al., 2020), and has been an active research area for over four decades (Izenman, 1975). MTL applies to a wide range of fields due to its fundamental nature. For example, Ghosn and Bengio (1996) used artificial neural networks (ANNs) to predict stocks investment profits over time. They considered 3,636 assets from Canadian large-capitalization stocks and from the Canadian treasury. A series of experiments showed a major improvement by allowing different levels of shared parameters among the targets. Other notable examples include chemometrics (Burnham et al., 1999), ecological modeling (Kocev et al., 2009), text classification (Schapire and Singer, 2000), and bioinformatics (Ji et al., 2008).

There are two main approaches for MTL. The first is typically referred to as *problem transformation methods* or *local methods*. It transforms the MTL problem into a series of single-target models, and single-output schemes are applied. The second approach is mostly known as *algorithm adaptation* or *global methods*. These methods train a single model simultaneously for all the targets. None of these approaches can universally outperform the other. Indeed, both have certain merits and limitations, as demonstrated in the following sections. The interested reader is referred to Adıyeke and Baydoğan (2020) for a thorough discussion.

Decision trees are among the most popular supervised learning schemes (Wu et al., 2008). Decision trees hold many favorable properties. They are simple to understand and interpret, able to handle numerical and categorical features and have the ability to capture non-linear and non-additive relationships. Training a decision tree typically requires recursive partitioning of the feature space into a set of rectangles. Several popular decision tree implementations were proposed over the years. For example, ID3 (Quinlan, 1986), CART (Li et al., 1984), C4.5/C5.0 (Quinlan, 2004, 2014) to name a few.

MTL has been utilizes to a variety of predictive models. In the context of decision-tree methods, there are two major MTL approaches. The first is to construct a single tree for each response variable (Kocev et al., 2009). The second is to train a joint tree for all response variables all together (De'Ath, 2002). A hybrid approach, which combines the two, is also considered in the literature (Santos et al., 2021; Alves and Cerri, 2022). In this work we introduce a new hybrid tree-based MTL framework. Specifically, we train decision trees that share some levels for all the targets, while allowing other levels to be target specific. Our proposed framework is motivated by the observation that both single and joint trees hold unique advantages in different scenarios. Single trees are advantageous in cases where the correlation between response variables are weak or non-existent, as they allow the flexibility to train more tailored models for each response variable. On the other hand, training a joint tree for all response variables can account for the relationship between the targets, if such exists. In this work we propose a hybrid approach that selects the appropriate method at each node by utilizing a cross-validation (CV) score. Specifically, the proposed approach determines whether to create a separate tree for each response variable or to build a joint tree for all response variables at each node based on its CV score. By combining the advantages of both schemes, our method adapts to the unique properties of the problem, resulting in improved predictive performance. Overall, our proposed hybrid approach offers a more flexible and effective solution compared to traditional methods. Our experiments demonstrate favorable performance compared to existing methods on various synthetic and real-world datasets. An implementation of the proposed method is publicly available.^1^

## 2 Related work

In this section we first overview existing multi-target algorithms. Next, we present the CART algorithm and describe the building process of the tree. Then, we introduce the ALOOF method, a novel approach to variable selection, which we adapt in our proposed framework. Finally, we discuss currently known multi-target tree-based algorithms.

### 2.1 Multi target learning

The MTL framework considers *n* independent observations from *p* features and *d* targets. Specifically, we denote the *i*^*th*^ observation as (*x*_*i*_, *y*_*i*_) where *x*_*i*_ = (*x*_*i*1_, *x*_*i*2_…*x*_*ip*_) and *y*_*i*_ = (*y*_*i*1_, *y*_*i*2_…*y*_*id*_). Notice that all *d* targets share the same set of features. As mentioned above, current MTL methods are typically either local or global, where each approach holds its own advantages and caveats.

#### 2.1.1 Local MTL methods

##### 2.1.1.1 The single target scheme

The most basic local approach is the baseline single target (ST) scheme (Spyromitros-Xioufis et al., 2016). Here, *d* separate models are learned for each target independently. Specifically, for response variable *r*, ST considers a training set $T_{r}={\left\{x_{i},y_{ir}\right\}}_{i=1}^{n}$ where *x*_*i*_ is the original feature vector *x*_*i*_ = (*x*_*i*1_, *x*_*i*2_…*x*_*ip*_).

##### 2.1.1.2 Stacked single target

Stacked Single Target (SST) (Spyromitros-Xioufis et al., 2016) is an MTL scheme for regression tasks, inspired by the multi-label classification method *Stacked Binary Relevance* (SBR) (Godbole and Sarawagi, 2004). The SST training process consists of two stages. First, *d* single models are separately trained for each response variable, as in ST. Then, *d* meta-models, one for each response variable, are trained in the second stage. Each meta-model is trained on a transformed training set $T_{r}^{\prime }={\left\{x_{i}^{\prime },y_{ir}\right\}}_{i=1}^{n}$, where $x_{i}^{\prime }=\left(x_{i1},\ldots ,x_{ip},{ŷ}_{i1},\ldots ,{ŷ}_{id}\right)$ is the original feature vector *x*_*i*_, augmented with the predictions from the first stage.

##### 2.1.1.3 Regressor and classifier chains

Regressor Chains (RC) (Spyromitros-Xioufis et al., 2016) and Classifier Chains (CC) (Read et al., 2011) train *d* models, similar in spirit to SST. Here, we first set a (random) order among the targets. Then, each target is trained on the predictions of the previous targets in the drawn order. For example, assume that *d* = 2 and the drawn order of targets is (*y*_2_, *y*_1_). Then, the training set for the first response variable *y*_2_ is $T_{2}^{\prime }={\left\{x_{i}^{\prime },y_{i2}\right\}}_{i=1}^{n}$, where $x_{i}^{\prime }$ is the original features vector. Next, we proceed to *y*_1_. The transformed training set for this target is $T_{1}^{\prime }={\left\{x_{i}^{\prime },y_{i1}\right\}}_{i=1}^{n}$ where now $x_{i}^{\prime }=\left(x_{i1},\ldots ,x_{ip},{ŷ}_{i2}\right)$ is the original feature vector, augmented with the ŷ_*i*2_ from the previous step.

#### 2.1.2 Global MTL methods

**Figure 1:**
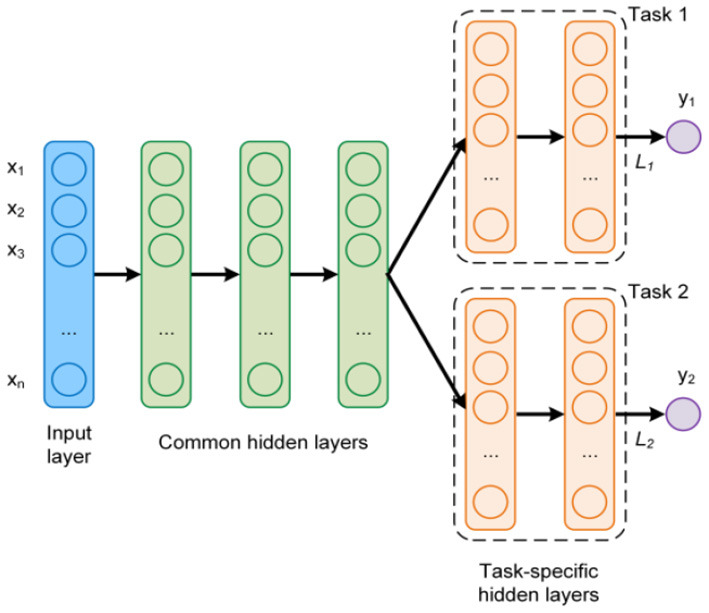
An example of a MTL network architecture with two targets (tasks).

Michelucci and Venturini (2019) proposed an MTL neural network architecture, consisting of common (joint) and individual hidden layers (see Figure 1). The common hidden layers consider all response variables simultaneously, as they strive to capture the dependencies among them. The outputs of these layers are used as inputs for the following individual layers. These, on the other hand, focus on the unique properties of each separate response and allow introduce flexibility to their proposed scheme.

Evgeniou and Pontil (2004) presented a different MTL method using a regularization approach. They focused on support vector machines (SVM) and extended this notion to the MTL setup. In SVM, the objective is to find a hyper-plane *w*^*T*^*x* − *b* = 0 with the largest distance to the nearest training data points of each class. Under the assumption that all targets' weight vectors *w* are “close to each other”, they defined the weight of the *r*^*th*^ target *w*_*r*_ as *w*_*r*_ = *w*_0_ + *v*_*r*_ where *w*_0_ is the mean of the *w*'s over all targets and *v*_*r*_ corresponds to the deviation from the mean. The objective function is similar to the single target scheme, with a summation of the parameters across all the targets. It contains two positive regularization parameters, for the two terms. The regularization parameters impose constraints and control the variability among the models.

Curds and Whey (C&W) is a procedure proposed by Breiman and Friedman (1997), for multiple linear regression with multivariate responses. C&W utilizes elements of canonical correlation and shrinkage estimation to enhance the prediction accuracy for each response variable. Specifically, C&W applies simple least squares regressions and then utilizes the correlations between the responses and features to shrink the predicted values from those regressions.

### 2.2 Classification and regression trees

As mentioned above, the focus of our work is the design of a decision tree-based MTL framework. For this purpose, we briefly review the popular Classification and Regression Tree (CART) algorithm. Consider *n* observations ${\left\{x_{i},y_{i}\right\}}_{i=1}^{n}$ consisting of *p* features, where *x*_*i*_ = (*x*_*i*1_, *x*_*i*2_…*x*_*ip*_) and *y*_*i*_ is a real (regression) or categorical (classification) scalar. During the training phase of the tree, CART performs recursive binary partitioning of the feature space. For each feature *j* we consider a collection of possible *split points* *S*_*j*_. Every split point *s* ∈ *S*_*j*_ corresponds to a binary partition of the *n* observation into two disjoint sets *L*(*s*) and *R*(*s*). For numerical/ordinal features the two sets define (without loss of generality) *L*(*s*) = {*X*|*X*_*j*_ < *s*} and *R*(*s*) = {*X*|*X*_*j*_ ≥ *s*}. Notice that in this case, |*S*_*j*_| < *n* as only unique values are considered along the sorted values. For categorical features the two sets are define as *L*(*s*) = {*X*|*X*_*j*_ ∈ *Q*_*jL*_} and *R*(*s*) = {*X*|*X*_*j*_ ∈ *Q*_*jR*_} where *Q*_*j*_ is the set of categories of variable *j* and *Q*_*jL*_, *Q*_*jR*_ are sub-sets such that *Q*_*jL*_ ∪ *Q*_*jR*_ = *Q*_*j*_ and *Q*_*jL*_ ∩ *Q*_*jR*_ = ∅. Here, there is a total of $|S_{j}|=2^{|Q_{j}|-1}-1$ possible binary splits. However, it is easy to show that one can order the categories by the corresponding mean of their response variables, and only consider the splits along this ordered list (Li et al., 1984). This leads to a total of |*Q*_*j*_|−1 candidate splits. For every split *s*∈*S*_*j*_, CART evaluates a loss criterion. In regression trees the popular choice is the squared loss,


(1) $$\begin{array}{l} L\left(s\right)=\Sigma_{i\in L\left(s\right)}{\left(y_{i}-{ȳ}_{L}\right)}^{2}+\Sigma_{i\in R\left(s\right)}{\left(y_{i}-{ȳ}_{R}\right)}^{2} \end{array}$$

where ȳ_*L*_ and ȳ_*R*_ are the mean over the sets *L*(*s*) and *R*(*s*), respectively. For a two-class classification tree it utilizes the Gini index loss criterion (Equation 2),


(2) $$\begin{array}{l} L\left(s\right)=n_{L}\hat{p_{L}}\left(1-\hat{p_{L}}\right)+n_{R}\hat{p_{R}}\left(1-\hat{p_{R}}\right) \end{array}$$

where *n*_*L*_, ${\hat{p}}_{L}$, *n*_*R*_, and ${\hat{p}}_{R}$ are the number of observations and the observed proportions of each of the classes in *L*(*s*) and *R*(*s*), respectively. Ultimately, CART seeks (j^*^, s^*^) that solve the minimization problem


(3) $$\begin{array}{l} \underset{\begin{array}{c} j\in \left\{1,\ldots ,p\right\}\\ s\in S_{j} \end{array}}{\min}L\left(s\right). \end{array}$$

Since CART is a recursive algorithm, it requires a stopping criterion to terminate the growth of the tree. Common criteria include a maximum depth, a minimum number of samples required for a split, a minimum number of samples at each leaf and a minimum decrease in loss. It is well-known that large trees tend to overfit the data (high variance and low bias) while smaller trees might not capture the all relationships between the features (high bias and low variance). A popular solution is by cross-validated pruning of the tree (Li et al., 1984).

### 2.3 Cross-validated trees

Large cardinally categorical features introduce a major statistical concern during the tree training process. Specifically, notice that CART tends to select variables with large |*Q*|, and consequently suffer from over-fitting. For example, consider a simple index feature. Here, Equation (3) would favor this feature over the alternatives, as it allows maximal flexibility in minimizing the objective. Recently, Painsky and Rosset (2016) introduced the *Adaptive Leave-one-out Feature Selection* scheme (ALOOF) to overcome this caveat. ALOOF suggests a new approach for variable selection as it ranks the features by estimating their generalization error. That is, the best-split is chosen based on its leave-one-out cross-validation performance (as opposed to the in-sample performance presented in Equation 3). As a result, ALOOF makes a “fair” comparison among the features, which does not favor features according to their cardinality.

### 2.4 Decision tree based MTL

One of the first MTL methods that consider decision trees was proposed by De'Ath (2002). In this work, the author introduced the concept of multivariate regression trees (MRTs). MRTs extend classical univariate regression trees (Li et al., 1984) to a multi-target setup. This requires redefining the loss criterion, as appears in Equation (1). Specifically,


(4) $$\begin{array}{l} \begin{array}{c} L\left(s\right)=\overset{d}{\underset{r=1}{\sum }}\underset{i\in L\left(s\right)}{\sum }{\left(y_{ir}-{ȳ}_{Lr}\right)}^{2}+\underset{i\in R\left(s\right)}{\sum }{\left(y_{ir}-{ȳ}_{Rr}\right)}^{2}, \end{array} \end{array}$$

where ȳ_*Lr*_ and ȳ_*Rr*_ are the means of the sets *L*(*s*) and *R*(*s*) for the *r*^*th*^ target. The training process of De'Ath (2002) is similar to standard CART, under the loss criterion above. Finally, each leaf of the tree stores *d* output values, which correspond to the mean of each response variable. A similar MTL extension to classification trees were also considered. Kocev et al. (2009) compared MRT with standard CART. Their results showed that MRT typically outperforms CART, despite no statistical significance in their results.

Piccart et al. (2008) suggested using a subset of response variables (denoted *support targets*) to predict a given “main” target. Notice that this goal is different than the classical MTL framework, which models on all targets. They proposed a local method, called *Empirical Asymmetric Selective Transfer (EAST)*. This model is based on the assumption that among the targets, some may be related while others are not. They argued that the related targets may increase the predictive accuracy, as opposed to the rest of the targets. To find the best support (related) targets for a given response variable *j*, EAST measures the increase in predictive performance that a candidate target yields using CV. The best candidate target is then added to the current support set. The algorithm returns the best support set that was found.

Basgalupp et al. (2021) presented a closely related method. They suggested alternative partitions of the response variables to disjoint sets. To find the best partitions they applied both an exhaustive search strategy, and a strategy based on a genetic algorithm. After finding the optimal subsets, the partition is treated as a separate prediction problem. They used decision trees and random forests as base models.

A multi-objective classifier called a *Bloomy Decision Tree* (BDT) was presented by Suzuki et al. (2001). The building tree process is similar to a classical CART decision tree. It recursively partitions the feature space based on an attribute selection function. The criterion they used for selecting the splitting point is the sum of gain ratios for each class. In the BDT, a *flower node* that predicts a subset of class dimensions is added to the tree. In order to select those class dimensions, at each internal node and for each class dimension, the algorithm employed pre-pruning based on Cramer's V (Weber, 1977). Unlike leaf nodes, flower nodes also appear in the internal nodes of the tree. Consequently, the number of class dimensions gradually decreases and we are able to circumvent the “fragmentation problem” (Salzberg, 1994).

Appice and Džeroski (2007) proposed an algorithm named *Multi-target Stepwise Model Tree Induction* (MTSMOTI). This method applies to regression problems, where leaves are associated with multiple linear models. At each step of tree construction, MTSMOTI either partitions the current training set (split node) or introduces a set of linear models. Here, each linear model corresponds to a response variable. The internal nodes contribute to capture global effects, while straight-line regressions with leaves capture only local effects.

The idea of combining local and global tree-based methods is also not new in the literature. Santos et al. (2021) introduce predictive bi-clustering trees (PBCT) for MTL. Their approach generalizes classical decision trees, where each node corresponds to bi-clustering of the data. That is, instead of splitting the data with respect to a feature (as in classical DT), the data is clustered with respect to both the features and the targets. This allows an exploitation of target correlations during the tree-building process. Unfortunately, such an approach is highly prone to overfitting, since bi-clustering introduces many degrees of freedom, compared to classical tree splitting. In addition, bi-clustering typically does not perform well in cases where the data is too imbalanced, enerating leaf nodes with a much higher number of negative interactions. This caveat was studied by Alves and Cerri (2022) who proposed a two-step approach, where PBCTs are used to generate partitions and an XGboost classifier is used to predict interactions based on these partitions. Osojnik et al. (2016) studied option predictive clustering trees (OPCT) for MTR. An OPCT is a generalization of predictive clustering trees, allowing the construction of overlapping hierarchical clustering (as opposed to non-overlapping clustering, such as in Santos et al., 2021; Alves and Cerri, 2022). This means that at each node of the tree, several alternative hierarchical clusterings of the subspace can appear instead of a single one. Additional variants and ensembles of predictive clustering trees were introduced by Breskvar et al. (2018), including bagging, random forests, and extremely randomized clustering trees. Finally, Nakano et al. (2022) discuss a deep tree-ensemble (DTE) method for MTL. This method utilizes multiple layers of random forest (deep forest), where every layer enriches the original feature set with a representation learning component based on tree-embeddings.

## 3 Methodology

Most Tree-based MTL frameworks strive to minimize the (overall) generalization error, $E\left(\overset{d}{\underset{r=1}{\sum }}l\left(Y_{r},f_{r}\left(X\right)\right)\right)$, where *l* is some loss function (for example, squared error in regression) and *f*_*r*_ is a tree-based model. As described in the previous section, there are two basic decision tree MTL approaches. The first is to train a single shared tree for all the targets simultaneously (*f*_*r*_ = *f*), while the second is to construct *d* separate *f*_*r*_ trees while allowing dependencies among them. Our suggested model merges these approaches and introduces a hybrid tree that capitalizes the advantages of both schemes.

### 3.1 The tree training process

**Figure 2:**
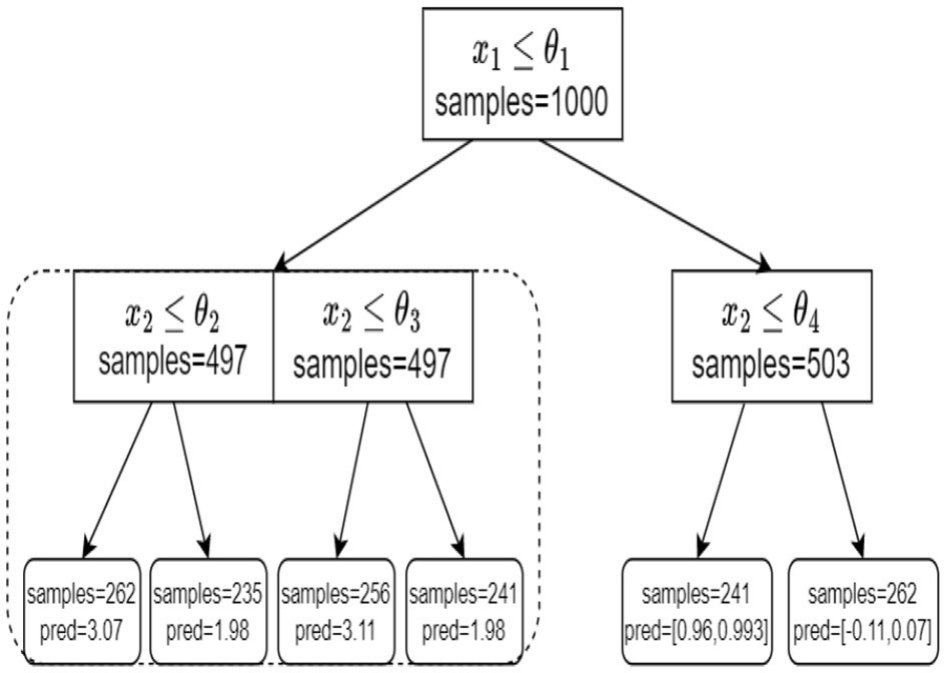
An example of our tree structure. At the root node, we split based on a single shared split. At the left child node, we treat each target independently. On the right node, we again split based on a single shared split.

We begin our tree training process in the following manner. First, we go over all *p* features and seek a single shared feature (and a corresponding split) for all the targets simultaneously. We evaluate the performance of the chosen split in a sense that is later described. Next, we evaluate the performance of every target independently. That is, for every target we seek a feature and a corresponding split value, independently of the other targets. We compare the two approaches and choose the one that demonstrates better results. Specifically, we choose whether to treat all the targets simultaneously with a single shared split (denoted as MT), or to treat each target independently, with its own split (like ST). To avoid extensive computation and statistical difficulties, we perform a no-regret tree growing process. This means that once we decide to split on each target independently, we do not go back to shared splits in consecutive nodes. The resulting model is a hybrid tree where higher levels are typically shared splits while deeper levels correspond to *d* independent trees (as illustrated in Figure 2). This hybrid tree follows the same rationale as the MTL neural network architecture in Figure 1.

### 3.2 Splitting criterion and evaluation

Naturally, one of the inherent challenges of our suggested method is to assess the performance of different splitting approaches (that is, MT vs. ST). Here, we follow the ALOOF framework (Section 2.3) and propose an estimator of the generalization error, based on cross-validation. Let $T={\left\{x_{i},y_{i}\right\}}_{i=1}^{m}$ be a set observations in a given node. For simplicity of the presentation, we first assume a regression problem where *y*∈ℝ^*d*^. Let *T*_*tr*_ and *T*_*val*_ be a partitioning of *T* into train and validation sets, respectively. Let *j* be an examined feature. Let $s_{j}^{*}$ be the optimal split value of the *j*^*th*^ feature over the train set. That is, $s_{j}^{*}$ is the argmin of Equation (4) over the set of observations *T*_*tr*_, while the corresponding loss is $L\left(s_{j}^{*}\right)$. We repeat this process for *K* non-overlapping partitioning of *T* to obtain *K* values of $L\left(s_{j}^{*}\right)$. Finally, we average these *K* results, similarly to a classical *K*-fold CV scheme. We denote the resulting average as


(5) $$\begin{array}{l} \begin{array}{c} GE_{MT}\left(j\right)=\frac{1}{K}\overset{K}{\underset{k=1}{\sum }}L^{\left(k\right)}\left(s_{j}^{*}\right) \end{array} \end{array}$$

where $L^{\left(k\right)}\left(s_{j}^{*}\right)$ is the loss of the *k*^*th*^ fold, as described above.

Next, we would like to estimate the generalization error of the ST splits. Here, we repeat the same process, but for every target independently. That is, for the *r*^*th*^ target and the *j*^*th*^ feature, we define *K* partitioning to train and validations sets. Then, we find the best split, $s_{j,r}^{*}$ over the train-set (following Equation 1), and evaluate its performance on the validation set. We repeat this process *K* times and average the results to obtain (Equation 6)


(6) $$\begin{array}{l} \begin{array}{c} GE_{ST}\left(j,r\right)=\frac{1}{K}\overset{K}{\underset{k=1}{\sum }}L^{\left(k\right)}\left(s_{j,r}^{*}\right) \end{array} \end{array}$$

where here $L^{\left(k\right)}\left(s_{j,r}^{*}\right)$ is the argmin of Equation (1) over the train-set, for the *r*^*th*^ target. Finally, we compare the optimal splitting choice when treating all targets simultaneously, $GE_{MT}^{*}=\underset{j}{\min}GE_{MT}\left(j\right)$ and treating each target independently, $GE_{ST}^{*}=\overset{d}{\underset{r=1}{\sum }}\underset{j}{\min}GE_{ST}\left(j,r\right)$. Algorithm 1 summarizes our proposed cross-validated splitting criterion. We continue the tree training process with the approach that yields the lower estimated generalization error. Specifically. if MT obtains a better result we proceed with a single shared split and repeat the process above for each of its child nodes. On the other hand, if ST is chosen we seek the optimal split for each of the *d* targets and proceed with a standard CART tree for each of the child nodes. We perform a no-regret tree-growing process, as previously described.

**Algorithm 1:** Comparing MT and ST.

**Input:** ${\left\{x_{i},y_{i}\right\}}_{i=1}^{m}$ a set of observations in a given node.
1: $GE_{ST}^{*}\leftarrow 0$.
2: for all *r* = 1 to *d* **do**
3: $GE_{ST}^{*}\leftarrow \underset{\begin{array}{c} j\in \left\{1,\ldots ,p\right\} \end{array}}{\min}GE_{ST}\left(j,r\right)+GE_{ST}^{*}$.
4: end **for**
5: $GE_{MT}^{*}\leftarrow \underset{\begin{array}{c} j\in \left\{1,\ldots ,p\right\} \end{array}}{\min}GE_{MT}\left(j\right)$.
6: return $\min\left(G\overset{*}{\underset{MT}{E}},GE_{ST}^{*}\right)$.

Cross-validation is a widely used approach for estimating the generalization capabilities of a predictive model. Specifically, in *K*-fold CV, the original sample is randomly partitioned into *K* equal-sized sub-samples. This allows all available information to be incorporated into the model training process, ensuring that no unique information is overlooked in the validation set. *K*-fold CV requires a choice of *K*, but it is unclear which value should be used. With ten-fold CV, the prediction error estimate is almost unbiased (Simon, 2007), so *K* = 10 is a reasonable off-the-shelf choice. Hence, we use the above throughout our experiments.

Finally, we need to consider a stopping criterion. For simplicity, we apply the popular CART grow-then-prune methodology. This approach involves initially growing a large tree and subsequently pruning it to achieve its favorable size through cross-validation. A pseudo-code of our proposed method is provided in Algorithm 2.

**Algorithm 2:** Our proposed method.

**Input:** ${\left\{x_{i},y_{i}\right\}}_{i=1}^{m}$ a set of observations in a given node.
1: Start at the root node.
2: Apply Algorithm 1 to find a split and use it to split the node into two child nodes.
3: if a stopping criterion is reached **then**
4: Exit.
5: else
6: if Splitting on multiple targets ($GE_{MT}^{*}<GE_{ST}^{*}$) is chosen in Step 2 **then**
7: Apply steps 2-11 to each child node.
8: else
9: Proceed with standard CART for each child node while treating each target independently.
10: end **if**
11: end **if**
12: Prune the tree with a CART pruning routine.

Although we focus our attention to regression trees, our proposed method can be easily applied to classification problems. Specifically, the only modification required is to replace the squared error with the Gini index (Equation 2). In fact, the Gini index is closely related to the squared error if we utilize with 0 − 1 coding for the classes (Painsky and Rosset, 2016).

### 3.3 Computational complexity

Having discussed the main components of our proposed framework, we turn to its computational complexity. In regression problems, CART first sorts the *n* observation pairs according to their feature values and determines a cut that minimizes the loss on both sides of the cut. By scanning along this list, *O*(*n*) operations are required, resulting in an overall complexity of *O*(*n*·*log*(*n*)) due to sorting. As previously noted, seeking a single shared split only extends the loss function, leading to the same complexity. On the other hand, seeking *d* separate splits requires *d* times CART complexity. Therefore, the overall computational load of our proposed method is *O*(*k*·*d*·*n*′·*log*(*n*′)), where *n*′ is the size of the train-set, *n*′ = (*k*−1)*n*/*k*. For classification, the only adjustment required is the replacement of the loss criterion. Hence, the computational complexity remains unaltered.

## 4 Experiments

Let us now demonstrate our proposed method in a series of synthetic and real worlds experiments.

### 4.1 Synthetic experiments

We begin with an illustration of our proposed method in a series of synthetic experiments. In the first experiment we draw 600 observations from two features and *d* targets, ${\left\{x_{i1},x_{i2},y_{i1},\ldots y_{id}\right\}}_{i=1}^{n}$. We define *X*_*ij*_ ~ *U*(−10, 10) and *r*^*th*^ target depends on the two features *X*_1_, *X*_2_ as follows.


$$Y_{r}=\{\begin{array}{ll} \epsilon_{r}, & \text{if }X_{1}>\alpha_{r}\text{ and }X_{2}>\alpha_{r}\\ 1+\epsilon_{r}, & \text{if }X_{1}>\alpha_{r}\text{ and }X_{2}<\alpha_{r}\\ 2+\epsilon_{r}, & \text{if }X_{1}<\alpha_{r}\text{ and }X_{2}>\alpha_{r}\\ 3+\epsilon_{r}, & \text{if }X_{1}<\alpha_{r}\text{ and }X_{2}<\alpha_{r} \end{array}$$

where ϵ_*r*_ ~ *N*(0, 1) i.i.d and α_*r*_ is a predefined parameter. Note that α_*r*_ determines the dependence between the features and the response variables. Further, notice that by choosing the α_*r*_'s very close to each other we get that the response variables are very correlated. Hence, in our experiments, we also use α_*r*_ as a parameter that controls the strength of the interaction between the response variables. The observations are split into 80% observations for the train-set and 20% for the test-set. We train the studied scheme on the train-set and evaluate the mean squared error (MSE) on the test-set. We further evaluate the ST and MRT as basic benchmarks. We repeat this process 500 times and report the averaged results.

**Figure 3:**
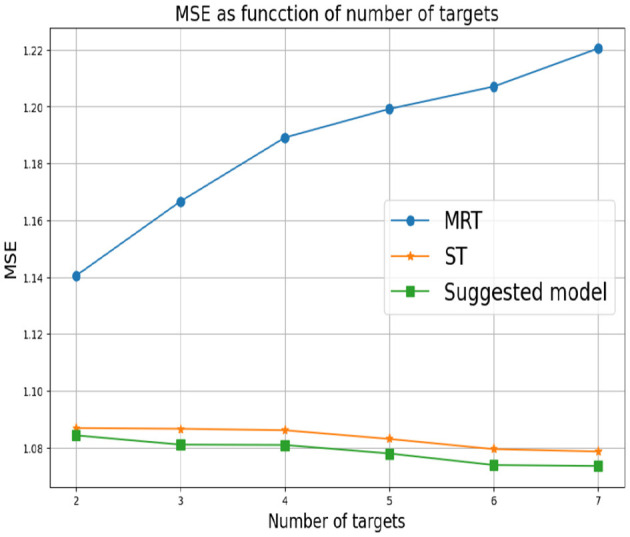
Synthetic experiment with two features and two response variables from the models are described in the text. The parameter α_1_ is set to zero and different values of α_2_ are evaluated.

**Figure 4:**
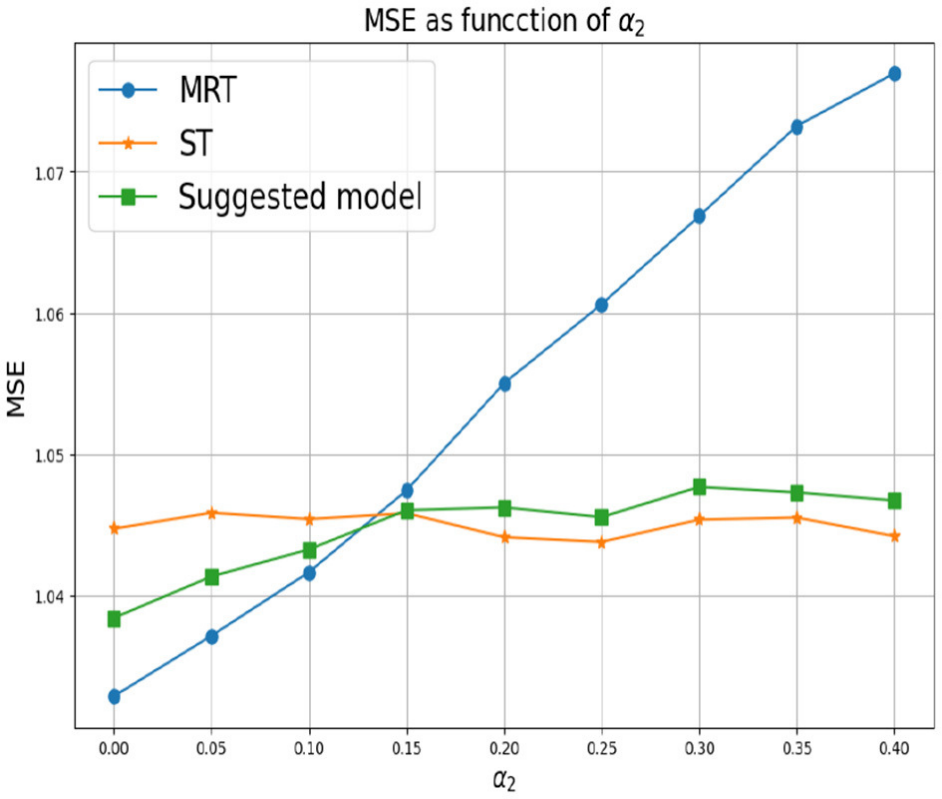
Synthetic experiment with two features. The parameters α_*r*_ are set to zero.

First, we set *d* = 2 which corresponds to two response variables. for *Y*_1_ we set α_1_ = 0 and for *Y*_2_ we consider different values of α_2_. As mentioned above, small values of α_2_ correspond to a greater correlation between the response variables. In this case, we expect MRT to be preferable. As α_2_ increases, the response variables become less related, so ST is the preferable choice. Figure 3 shows that our proposed method successfully tracks the preferred approach in both cases. Specifically, for smaller α_2_ we obtain a single tree with typically two levels, corresponding to the four possible outputs of the response variables. As α_2_ increases we typically obtain two separate trees, where each tree corresponds to the four (different) outputs of each target. Next, we examine the effect of the number of response variables *d*. We set the values of α's to zero for each response variable, indicating that the response variables are derived from the same model and are strongly dependent. Figure 4 demonstrates the resulting MSE as the number of response variables increases. The upper curve corresponds the ST approach, which is agnostic to the number of response variables and the underlying models. The lower curve corresponds to the MRT approach, which demonstrates superior performance as the number of response variables increases due to their strong correlation. The middle curve is our proposed method, which successfully tracks the preferred MRT approach and enhances its accuracy as the number of response variables grows. Once again, our proposed method typically outputs a single tree with four output level, corresponding to α = 0 as desired.

**Figure 5:**
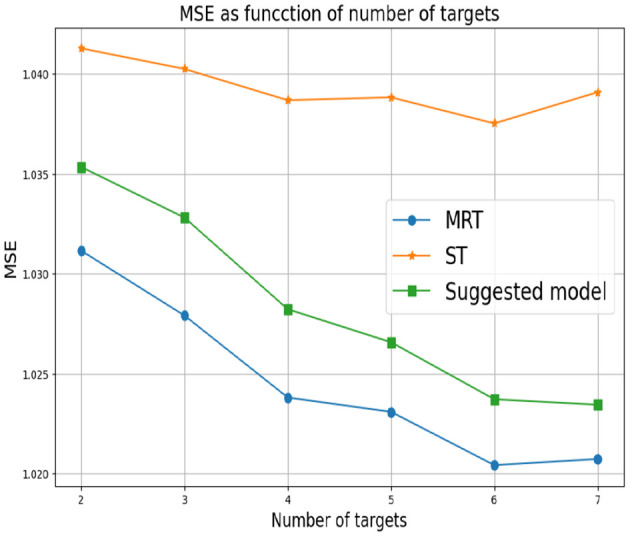
Synthetic experiment with two features. The parameters α_*r*_ are randomly drawn.

In the third experiment, the α_*r*_'s are arbitrarily chosen. This means that the response variables are derived from different models and there is an unknown dependence between them. Figure 5 summarizes the results we obtain. Here, MRT demonstrates a reduction in performance as the number of response variables increases. This decline can be explained by MRT attempting to exploit non-existent dependencies. This adverse effect becomes more pronounced as the number of response variables increased. As in the previous experiment, the ST approach at the bottom is agnostic to the number of response variables. However, it achieves superior performance in this setup, as the responses are (more likely) uncorrelated. Once again, our proposed method successfully tracks the favorable approach.

### 4.2 Real world experiments

We now turn to a real-world comparative study. Here, we not only demonstrate our approach in different setups but also compare it to additional alternatives. In the following experiments, we compare our proposed method with the standard ST and MRT schemes as above. In addition, we evaluate a model selection approach which utilizes CV to identify the best model among the two (that is, chooses between ST and MRT). We denote this scheme as *ST/MRT*. Furthermore, we implement RC/CC and SST/SBR (for regression and classification problems, respectively), with CART as a base model. See Section 2.1.1 for a detailed discussion. We also compare our proposed method with clustering trees (Breskvar et al., 2018) and deep tree-ensembles (DTE) (Nakano et al., 2022). Specifically, we apply the ROS-based methods in Breskvar et al. (2018) and the three deep forest schemes proposed by Nakano et al. (2022), denoted as X TE, X OS and X TE OS. Additional tree-based MTL methods are omitted as they focus on different merits (Piccart et al., 2008), or do not offer a publicly available implementation (and are too complicated to implement and tune) (Suzuki et al., 2001; Basgalupp et al., 2021). In addition, we increase the scope of our study and consider a *Gradient Boosting* (GB) framework (Friedman, 2001). That is, we implement a GB framework where the sub-learners are either MT, ST, or our proposed method. As the common practice, we implement GB with tree models and refrain from complex sub-learners (such as SST/SBR and RC/CC).

MTL has been extensively studied over the years, with several publicly available datasets. In the following, we briefly describe them and summarize their main properties. All these datasets are publicly available on openML^2^ and Kaggle.^3^ In the Scpf dataset, we predict three targets that represent the number of views, clicks, and comments that have been collected from US major cities (Oakland, Richmond, New Haven, and Chicago). The dataset includes seven features such as the number of days the issue stayed online, the source of the issue (e.g., android, iPhone, remote API), the issue type (e.g., graffiti, pothole, trash), geographical coordinates of the issue, the city it was published from, and the distance from the city center. All multi-valued nominal variables were converted to binary, and rare binary variables (<1 of the cases) were removed. The focus of the Concrete Slump dataset (Yeh, 2007) is to predict the values of three concrete properties, namely slump, flow, and compressive strength, based on the composition of seven concrete ingredients, which include cement, fly ash, blast furnace slag, water, superplasticizer, coarse aggregate, and fine aggregate. The Jura dataset (Goovaerts, 1997) comprises measurements of seven heavy metals (cadmium, cobalt, chromium, copper, nickel, lead, and zinc) taken from locations in the topsoil of the Swiss Jura region. Each location's type of land use (Forest, Pasture, Meadow, Tillage) and rock type (Argovian, Kimmeridgian, Sequanian, Portlandian, Quaternary) were also recorded. The study focuses on predicting the concentration of three more expensive-to-measure metals (primary variables) using cheaper-to-sample metals (secondary variables). The response variables are cadmium, copper, and lead, while the remaining metals, land use type, rock type, and location coordinates serve as predictive features. Overall we utilize 1,515 features for prediction. Finally, the E-Commerce dataset comprises transaction records spanning the period from March to August 2018. The dataset contains several features, including the customer's ID, the category name, and the grand total, which represents the amount of money spent on each transaction. Prior to analysis, we preprocessed these features to create a new dataset, where each column represents a specific category and each row corresponds to a specific customer and the amount of spending on each category. We focus on “Mobiles & Tablets” and “Beauty & Grooming” as response variables. Consequently, the remaining 1,414 categories are treated as features in our analysis. Moreover, we analyze only those customers who had made purchases in a minimum of nine categories, to avoid the issue of sparse data. Furthermore, we examine our proposed method for classification. Specifically, we convert SCPF and E-Commerce to two-class classification problems by comparing their target values with their medians. In addition to the above, we study several benchmark datasets which are popular in the MTL literature. Their detailed descriptions are provided in Melki et al. (2017); Breskvar et al. (2018); Nakano et al. (2022), for brevity.

To evaluate the performance of the suggested method, we use MSE for regression and 0 − 1 loss for classification (Painsky, 2023). For GB we utilize 50 trees and limit their complexity by defining the minimum number of observations in the trees' terminal nodes to be 0.05·*n*. To ensure that our results are robust and are not influenced by the particular random partitioning of the data we apply standard ten-fold CV. It is important to emphasize that for each dataset, different targets may have a different scale. This leads to bias toward large scale targets. To overcome this difficulty, we normalize the targets accordingly.

Tables 1, 2 summarize the results we achieve for a single tree and an ensemble of models, respectively. For each experiment we report the averaged merit and its corresponding standard deviation in parenthesis. For each dataset, we mark (with a bold font) the method that achieves the best averaged performance. As we can see, our proposed method demonstrates superior accuracy for a single tree, while the difference is less evident with ensembles. This highlights the well-known advantage of using ensemble methods over a single tree, as they can mitigate the limitations of a single tree. Nevertheless, we also observe an evident improvement in the ensemble setup. To validate the statistical significance of our results, we apply a standard sign test (Demšar, 2006) between our proposed method and each of the alternatives. Specifically, we count the number of datasets in which our proposed method defeats each alternative scheme. Then, we test the null hypothesis that both methods perform equally well. We report the corresponding *p*-values for each alternative method. For single tree models we obtain *p*-values of 0.0014, 0.0195, 0.0058 when tested against ST, MRT and ST/MRT, respectively. These results imply that even with an appropriate multiplicity correction for three hypotheses, our proposed method is favorable with a statistical significance level of 0.0585. For ensemble models we obtain *p*-values of 0.0005, 0.0005, 0.0195, 0.0058, 0.0058, 0.0058, 0.0541, and 0.0195 when tested against SST/SBR, RC/CC, X TE, X OS, X TE OS, GB-ST, GB-MRT and GB-ST/MRT, respectively. Once again, we observe relatively low *p*-values which emphasize the validity of our results. Yet, these findings are less significant (after appropriate multiplicity correction), due to the greater number of alternative methods. In addition, we compare our proposed method to Breskvar et al. (2018), who focused on the aRMMSE measure (see Equation 5 in Breskvar et al., 2018). We repeat the experiments above and evaluate the aRMMSE for the last four datasets in Table 2, which were also studied in Breskvar et al. (2018). Our proposed method outperforms (Breskvar et al., 2018) in all of these datasets. To conclude, our results introduce favorable results over the alternative schemes, where the advantage is more evident in the more interpretable single tree setup.

**Table 1:** Real-world data experiments non-ensembles.

**Data**	**ST**	**MRT**	**ST/MRT**	**Our method**
Slump	0.057 (0.076)	0.058 (0.076)	0.057 (0.077)	**0.049** (0.075)
Jura	0.0146 (0.0042)	0.0154 (0.0045)	0.0146 (0.0044)	**0.0142** (0.0038)
Scpf	0.00188 (0.0025)	0.00183 (0.0024)	0.00189 (0.0026)	**0.00181** (0.0026)
E-commerce	0.00261 (0.0026)	0.00261 (0.0026)	0.00269 (0.0026)	**0.00252** (0.0028)
Scpf classification	0.0981 (0.065)	0.1191 (0.087)	**0.0897** (0.04)	0.0979 (0.066)
E-commerce classification	0.3528 (0.029)	0.3611 (0.025)	0.3539 (0.031)	**0.3521** (0.027)
edm	0.0394 (0.0113)	**0.0373** (0.0123)	0.0394 (0.0113)	0.00374 (0.0114)
sf1	0.0261 (0.0131)	0.0251 (0.0139)	0.0261 (0.0131)	**0.0250** (0.0139)
sf2	0.00553 (0.00225)	0.00508 (0.00206)	0.00553 (0.00225)	**0.00506** (0.00188)
wq	0.06098 (0.00361)	0.06088 (0.00365)	**0.06088** (0.00361)	0.06155 (0.00343)
andro	0.0223 (0.02331)	0.0522 (0.02331)	0.0223 (0.02331)	**0.0222** (0.02248)
osales	0.00552 (0.00163)	0.00551 (0.00208)	0.00552 (0.00163)	**0.00550** (0.00137)
Friedman	0.0735 (0.00658)	**0.0732** (0.00577)	0.0735 (0.00658)	0.0745 (0.0067)
Music
Origin	0.0389 (0.00539)	0.0381 (0.00384)	0.0389 (0.00539)	**0.0380** (0.00450)
Polymer	**0.0122** (0.01202)	0.166 (0.01871)	0.0122 (0.01202)	0.0155 (0.01565)
atp1d	0.0106 (0.00319)	0.01337 (0.00513)	0.01061 (0.00319)	**0.00978** (0.00265)
atp7d	0.001197 (0.0075)	0.001397 (0.00799)	0.01197 (0.00798)	**0.01157** (0.00848)
oes97	0.00481 (0.00243)	**0.00435** (0.00268)	0.00481 (0.00243)	0.00474 (0.00256)
oes10	0.00272 (0.00170)	0.00412 (0.00377)	0.00412 (0.00365)	**0.00267** (0.00177)

Bold values indicate the best performing method.

**Table 2:** Real-world data experiments—ensembles.

**Data**	**SST/ SBR**	**RC/CC**	**X TE**	**X OS**	**X TE OS**	**GB ST**	**GB MRT**	**GB ST/MRT**	**GB ourmethod**
Slump	0.051 (0.08)	0.057 (0.079)	0.0568 (0.02262)	0.0430 (0.0266)	0.0450 (0.0302)	0.043 (0.067)	0.042 (0.07)	0.043 (0.07)	**0.038** (0.056)
Jura	0.0152 (0.044)	0.0152 (0.043)	0.0117 (0.00397)	0.0109 (0.00390)	0.0133 (0.00445)	**0.0103** (0.003)	0.0108 (0.004)	0.0103 (0.033)	0.0105 (0.035)
Scpf	0.00179 (0.0026)	0.00189 (0.0026)	**0.00151** (0.00026)	0.00168 (0.00103)	0.00161 (0.0009)	0.00169 (0.0016)	0.00168 (0.0014)	0.00168 (0.0014)	0.00167 (0.0017)
E-commerce	0.00272 (0.0029)	0.00269 (0.0026)	0.001724 (0.00017)	0.00173 (0.00016)	**0.001683** (0.00016)	0.00181 (0.0026)	0.00181 (0.0026)	0.00181 (0.0026)	0.0018 (0.0025)
Scpf classification	0.1116 (0.072)	0.0897 (0.04)	0.0831 (0.00297)	0.0910 (0.00231)	0.0796 (0.00247)	0.0814 (0.0163)	**0.0768** (0.0179)	0.0815 (0.0163)	0.0777 (0.0187)
E-commerce classification	0.3937 (0.049)	0.3539 (0.031)	0.335 (0.0018)	0.381 (0.00258)	0.0328 (0.00138)	0.3196 (0.0392)	0.322 (0.0394)	0.3196 (0.0392)	**0.3132** (0.0393)
edm	0.0397 (0.0137)	0.0379 (0.0125)	0.0374 (0.0092)	0.0359 (0.0121)	0.0359 (0.0151)	0.0351 (0.0139)	0.0344 (0.132)	0.0351 (0.0139)	**0.0321** (0.0134)
sf1	0.0259 (0.0131)	0.0261 (0.0134)	0.0291 (0.0089)	0.0283 (0.0017)	0.0318 (0.0091)	0.0265 (0.0141)	0.0261 (0.0141)	0.0265 (0.0143)	**0.0245** (0.0135)
sf2	0.00547 (0.0022)	0.00564 (0.00236)	0.00503 (0.00189)	0.00498 (0.0021)	0.00504 (0.00203)	0.00503 (0.00178)	0.00503 (0.00183)	0.00503 (0.00178)	**0.00495** (0.00188)
wq	0.06116 (0.00374)	0.06174 (0.00397)	0.05539 (0.00282)	0.05903 (0.00375)	0.05909 (0.00341)	0.5569 (0.00249)	0.05448 (0.00248)	0.05569 (0.00249)	**0.05434** (0.00262)
andro	0.0185 (0.02545)	0.0256 (0.03037)	0.0285 (0.02869)	0.0207 (0.02082)	0.0200 (0.02140)	0.0172 (0.02577)	**0.0089** (0.01116)	0.0172 (0.02577)	0.0129 (0.01861)
osales	0.00556 (0.00182)	0.00607 (0.00241)	**0.00492** (0.00132)	0.00494 (0.00131)	0.00494 (0.00143)	0.00523 (0.00179)	0.00523 (0.00171)	0.00499 (0.00165)	0.00527 (0.00177)
Friedman	0.0733 (0.00663)	0.0737 (0.00616)	**0.0729** (0.00567)	0.0907 (0.00787)	0.0981 (0.00714)	0.0774 (0.00658)	0.737 (0.00544)	0.0774 (0.00715)	0.0730 (0.00571)
Music Origin	0.0391 (0.00563)	0.0626 (0.00827)	0.0318 (0.00397)	**0.0304** (0.00388)	0.0329 (0.00395)	0.0310 (0.00388)	0.0303 (0.00398)	0.0310 (0.00388)	0.0306 (0.00370)
Poly.	0.0107 (0.0114)	0.0137 (0.0153)	0.0169 (0.02561)	0.0161 (0.0221)	0.0152 (0.02291)	0.00781 (0.01187)	0.00873 (0.01267)	**0.00781** (0.01187)	0.00857 (0.01366)
atp1d	0.01068 (0.00349)	0.00923 (0.00398)	0.0082 (0.0024)	0.00809 (0.00236)	0.00831 (0.00263)	0.00646 (0.00207)	0.00679 (0.00257)	0.00646 (0.00207)	**0.00644** (0.00203)
atp7d	0.01279 (0.00783)	0.01279 (0.00753)	0.00765 (0.00614)	0.00796 (0.00514)	0.00812 (0.00613)	**0.00742** (0.00595)	0.00737 (0.00531)	0.00742 (0.00595)	0.00762 (0.00614)
oes97	0.0044 (0.00183)	0.00474 (0.002202)	0.00396 (0.00145)	0.0042 (0.00141)	0.00386 (0.00097)	0.00387 (0.00255)	0.00395 (0.00255)	0.00377 (0.00237)	**0.00376** (0.00255)
oes10	0.00274 (0.00160)	0.00286 (0.00173)	**0.00207** (0.00109)	0.00221 (0.00100)	0.00215 (0.00109)	0.00258 (0.00226)	0.00269 (0.00229)	0.00244 (0.00213)	0.00258 (0.00227)

Bold values indicate the best performing method.

Finally, we evaluate and compare the execution time of the studied methods. Our proposed method takes ~2-3 times more to apply, on the average, then the traditional CART (that is, without ensembles). The reason that the computational load is less than a factor of ten (as one may expect from our worst-case analysis in Section 3.3) is quite straightforward. Our proposed method begins with a 10-fold CV in each level of the tree. However, once we observe that independent trees become favorable (in terms of expected generalization error), we continue the tree construction with traditional CART (see Step 9 of Algorithm 2).

## 5 Conclusions

In this work we propose a novel tree-based model for MTL. Our suggested framework utilizes the advantages of ST and MRT as we introduce a hybrid scheme of joint and separate splits. By adopting a CV framework for selecting the best approach at each node, we minimize the (estimated) generalization error to avoid overfitting and improve out-of-sample performance. We demonstrate our suggested approach in synthetic and real world experiments, showing preferable merits over alternatives.

Our work emphasizes the importance of carefully considering the trade-offs between joint and separate modeling when designing MTL methods. By identifying the strengths and weaknesses of both approaches and combining them in an innovative way, we achieve results that surpass those of both decision tree and gradient boosting methods. These findings have important implications for the development of more robust and versatile machine learning algorithms. Our method offers a promising solution to the challenge of MTL. It provides an effective approach to optimize performance while maintaining interpretability, critical factors for practical applications.

## Data availability statement

Publicly available datasets were analyzed in this study. The datasets can be found in the OpenML and/or Kaggle repositories, and can be accessed via the following links: https://www.openml.org/search?type=data&sort=runs&id=41555&status=active; https://www.openml.org/search?type=data&sort=runs&id=41558&status=active; https://www.openml.org/search?type=data&status=active&id=41554&sort=runs; https://www.kaggle.com/datasets/zusmani/pakistans-largest-ecommerce-dataset.

## Author contributions

YN: Data curation, Methodology, Software, Validation, Writing – original draft. AP: Conceptualization, Formal analysis, Funding acquisition, Investigation, Methodology, Project administration, Resources, Supervision, Validation, Writing – review & editing.

## Footnotes

^1^ https://github.com/a4566201/-Cross-validated-Tree-Based-Models-for-Multi-target-Learning

^2^ https://www.openml.org/

^3^ https://www.kaggle.com/
